# Supplementary material for: Nanoscale profiling of evolving intermolecular interactions in ageing FUS condensates
Source: Commun Chem. 2025 Sep 29;8:284. doi: 10.1038/s42004-025-01659-z (PMC12479745; doi:10.1038/s42004-025-01659-z)
Supplement: Supplementary file 2 — Supplemental Information [file 42004_2025_1659_MOESM2_ESM.pdf]

Supplementary Information for:

Nanoscale profiling of changing intermolecular interactions in ageing FUS condensates

Supplementary Note 1: Evaluation of the contact model

Force-distance curves are converted to Young's Modulus values via the built-in Nanoscope Analysis software using the Derjaguin-Muller-Toporov (DMT) model. This an extension of the Hertz model:

$$F_H(\delta) = \frac{4\sqrt{R}}{3(1-\upsilon^2)} E\delta^{3/2}$$

that considers additional contributions from adhesion outside the contact area (*1*),

$$F_{DMT}(\delta) = F_H(\delta) - 2\pi Rw$$

with F = force, δ = indentation, R = radius, v = Poisson's ratio (approximated to 0.5 for biological samples), E = Young's Modulus, w = work of adhesion.

The DMT model is a commonly applied contact model in biological studies of relatively stiff material with low adhesion, as shown in Figure 4b (*2*–*6*). However, this model relies on the assumption that the sample is an elastic half-space. This assumption rarely holds in the case of biological specimens, thus introducing an inherent error in the reported Young's modulus values, which we minimise by ensuring indentation depth of ~10% of the height of the sample (according to Buckle's rule) and only considering in our analysis large condensates. In these experimental conditions, we estimate this assumption introduces an error of 8% (*7*), which is minimal compared to the inherent error of nanomechanical measurements which is at least 30%.

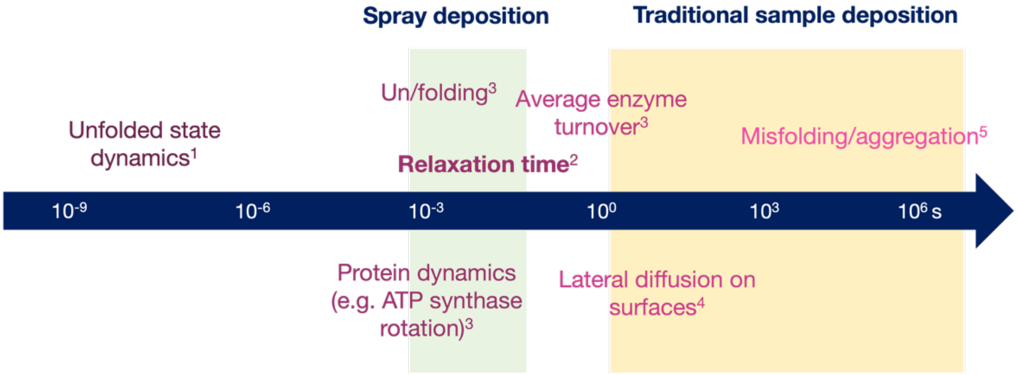

Figure S1. Timescales of protein dynamics. The timescales of drying in spray deposition versus manual deposition are contextualised by considering timescales of protein dynamics. Spray deposition times are comparable to reported relaxation times of biomolecular condensates. Values taken from the supplementary references (*8*–*12*).

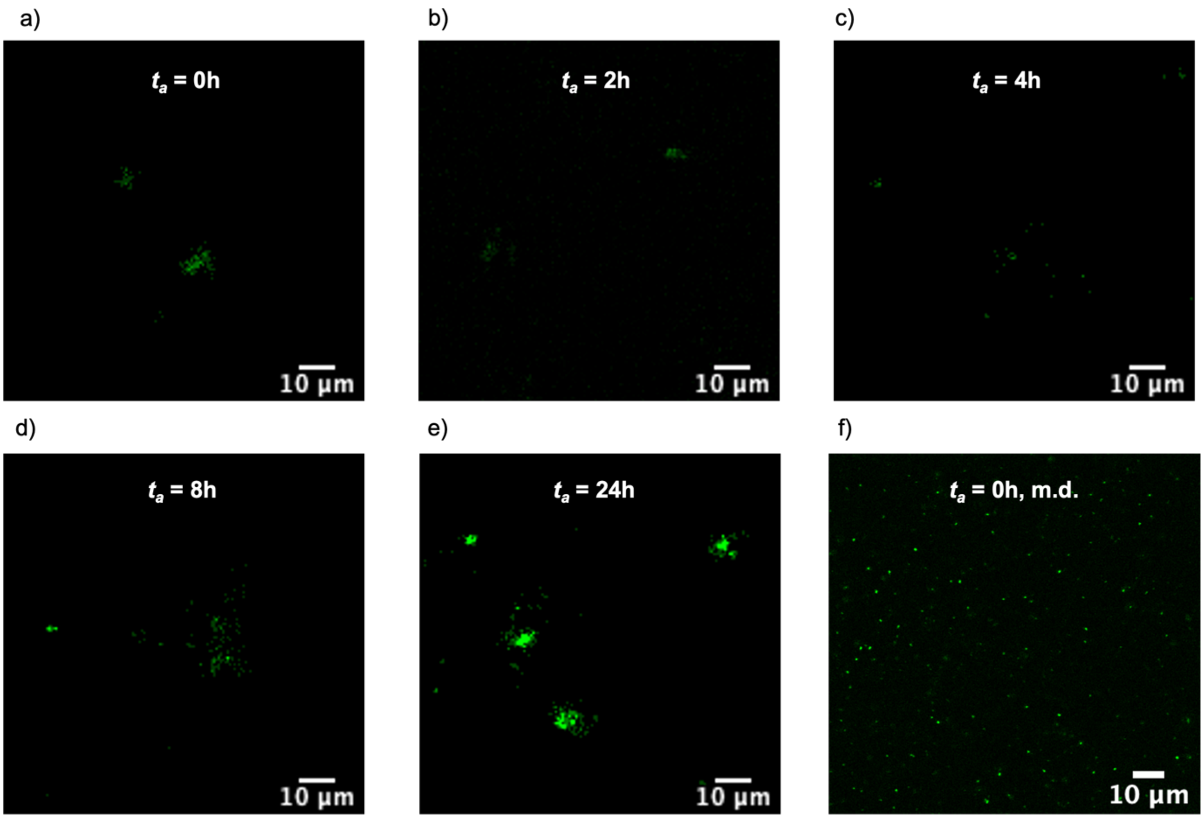

Figure S2. Confocal images of FUS condensates at various ageing times. (a-e) Condensates deposited via microfluidic spray were imaged at t_a = 0 (a), 2 (b), 4 (c), 8 (d), and 24 h (e). Very few, faint condensates are observed at early ageing times (t_a = 0, 2 and 4 h), and more, brighter condensates are observed at late ageing times (t_a = 8 and 24 h). **(f)** Samples were also imaged in solution, deposited via pipetting.

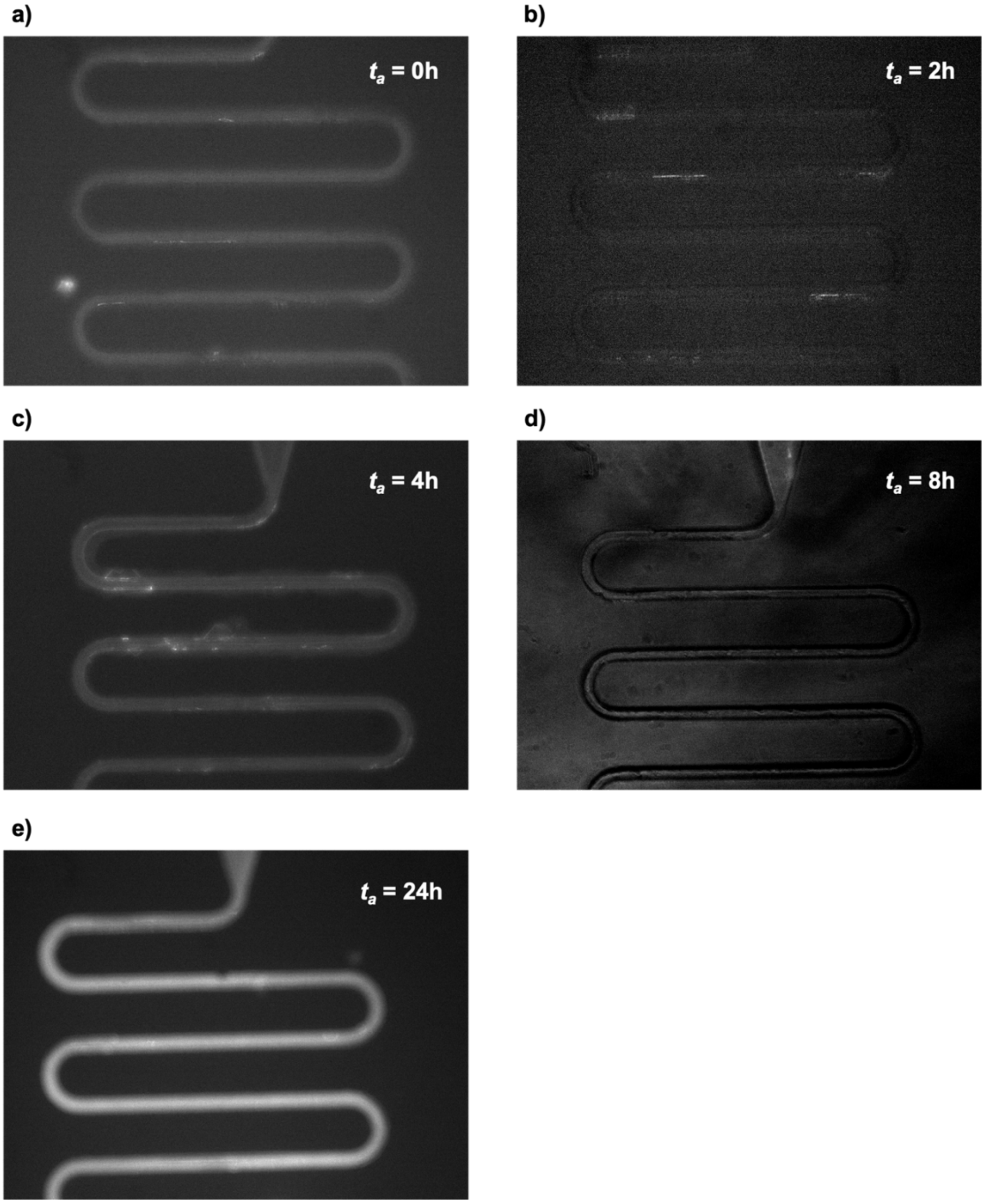

Figure S3. Fluorescent images of FUS deposition within microfluidic device channels. (a-e) Channels of the microfluidic spray devices were imaged after sample deposition to assess the extent of bio-fouling. Low levels of fluorescence intensity at early time points, t_a = 0 h (a), and 2 h (b) indicate little material is lost in the device. At a late time-point, t_a = 24 h (e), there is higher fluorescence intensity, indicating that some material is retained in the channels.

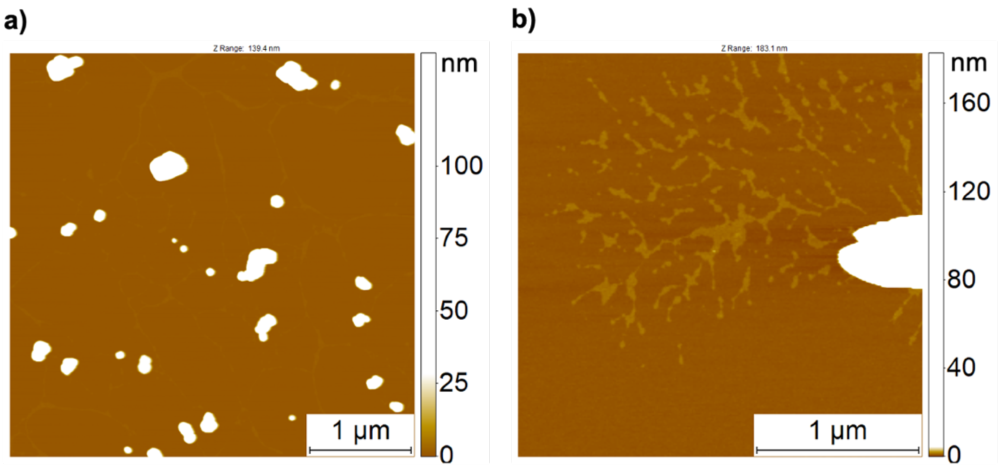

Figure S4. AFM imaging of deformation of FUS condensates upon deposition on mica surfaces. (a-b) Sample deformation was observed when FUS condensates were deposited on charged mica substrate. Condensates appeared to have amorphous conformations (a) and appeared to wet the surface (b).

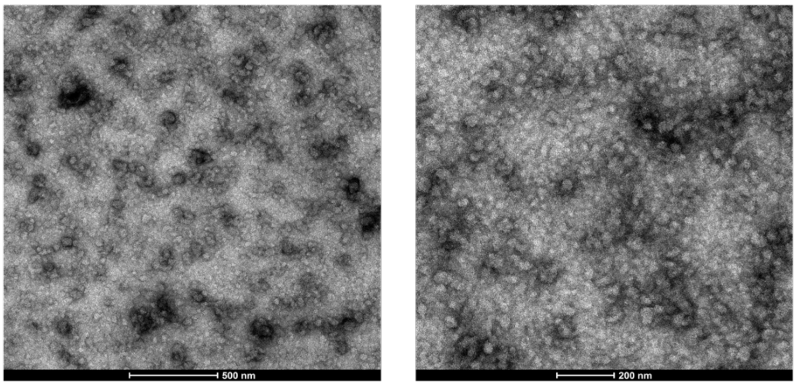

Figure S5. TEM images of solid-like aged FUS condensates. Condensates were deposited and imaged via TEM at t_a = 24 h.

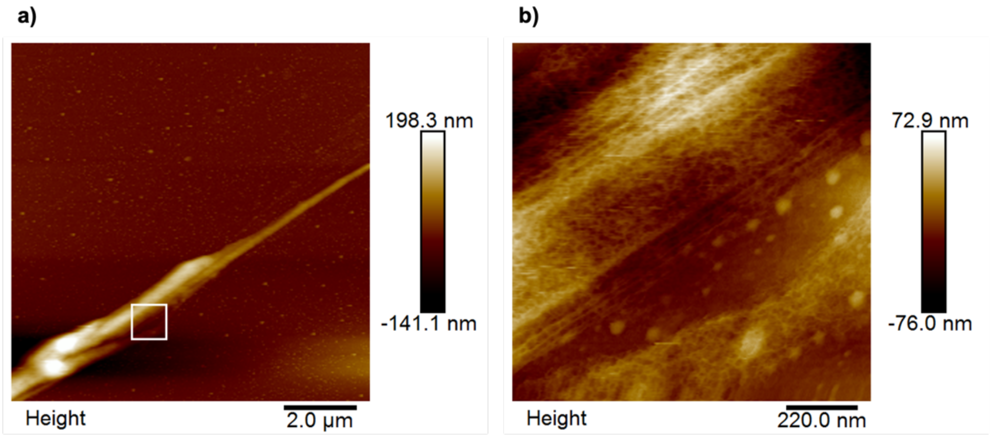

Figure S6. Micron-scale fibrous structures form from FUS solutions. (a,b) AFM images of elongated structures when aged condensates (t_a=24 h) were deposited via manual deposition. A zoom of the structure in (a) is indicated by a white square. High-resolution imaging reveals a mesh-like network in these fibrous structures (b).

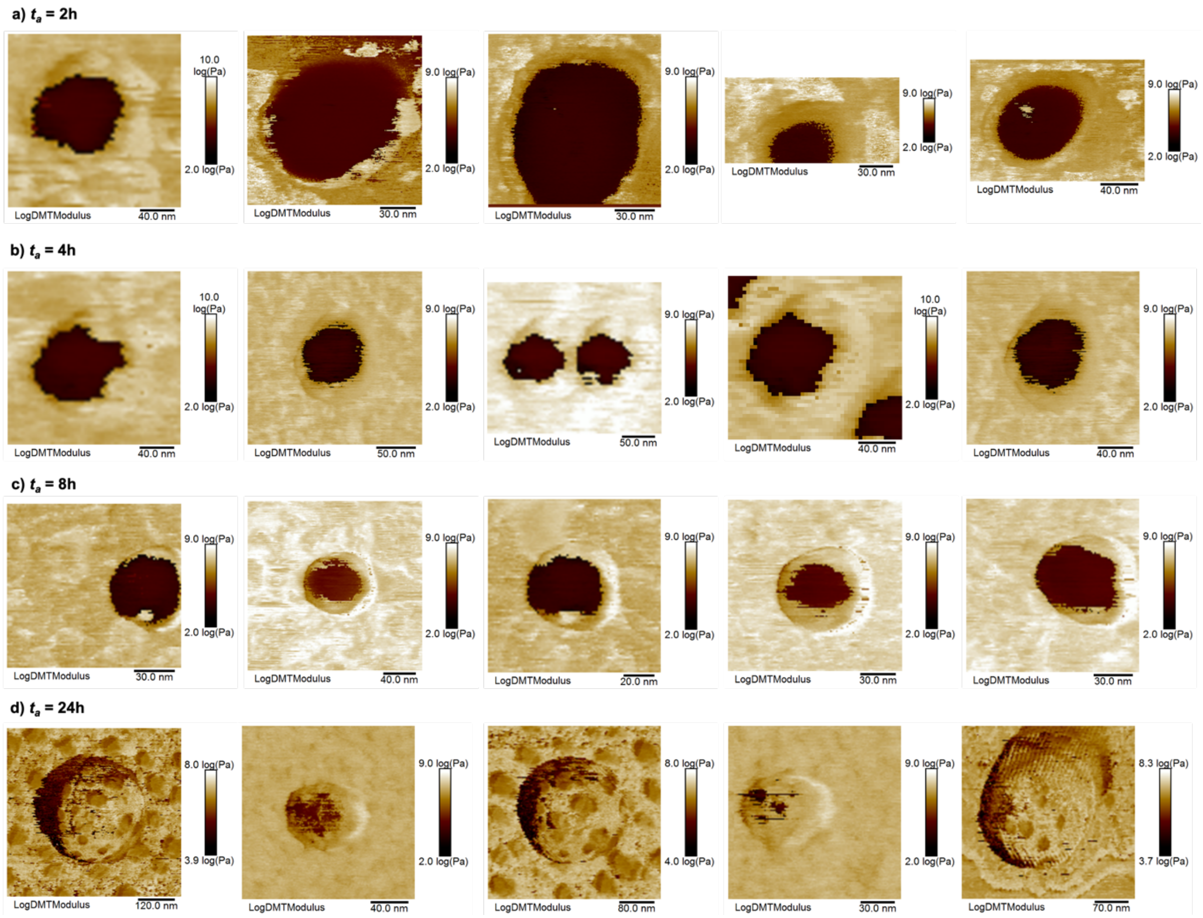

Figure S7. Nanomechanical maps of condensates. (a-d) Additional representative images, acquired via nanomechanical mapping, are shown of condensates as a function of t_a = 2 h (a), 4 h (b), 8 h (c) and 24 h (d).

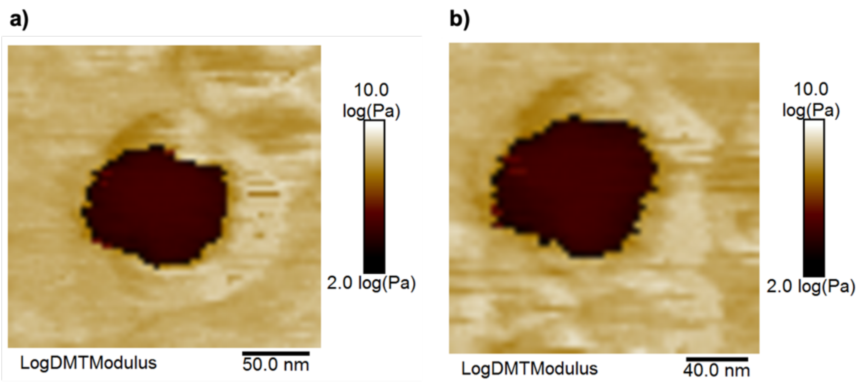

Figure S8. Nanomechanical imaging repeated on the same condensate. (a,b) Condensates were imaged (a), then re-imaged (b) to assess whether nanomechanical characterisation altered the morphology or nanomechanical properties. No detectable differences were observed when condensates were re-imaged.

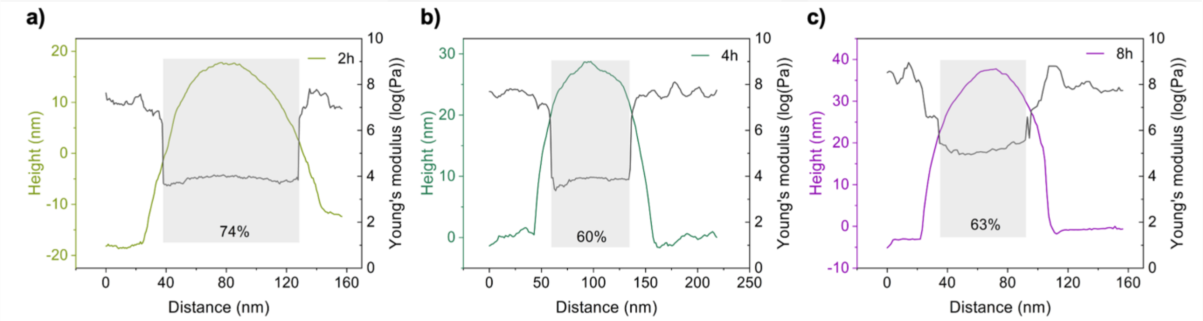

Figure S9. Solid phase formation at condensate-solvent interfaces. (a-c) Representative cross-sections of the height and Young's modulus for condensates at t_a=2 h (a), 4 h (b) and 8 h (c), taken at the centre of condensates. The percentage of the low elastic modulus phase for each condensate was calculated by considering the Young's modulus cross-section vs the condensate width (from the height profile). If the observed high elastic modulus phase emergence at condensate-solvent interfaces were solely due to the edge effect artefact, then the percentage of the low modulus phase would be smaller for softer condensates at t_a = 2 h. However, the opposite is observed, with a decrease in the low modulus percentage at later aged condensates (t_a = 4 and 8 h). We can also observe from these cross-sections that the high-modulus shell is lower than the surface alone. This indicates that the observation of a high modulus, solid-like shell is not solely due to the edge effect artefact.

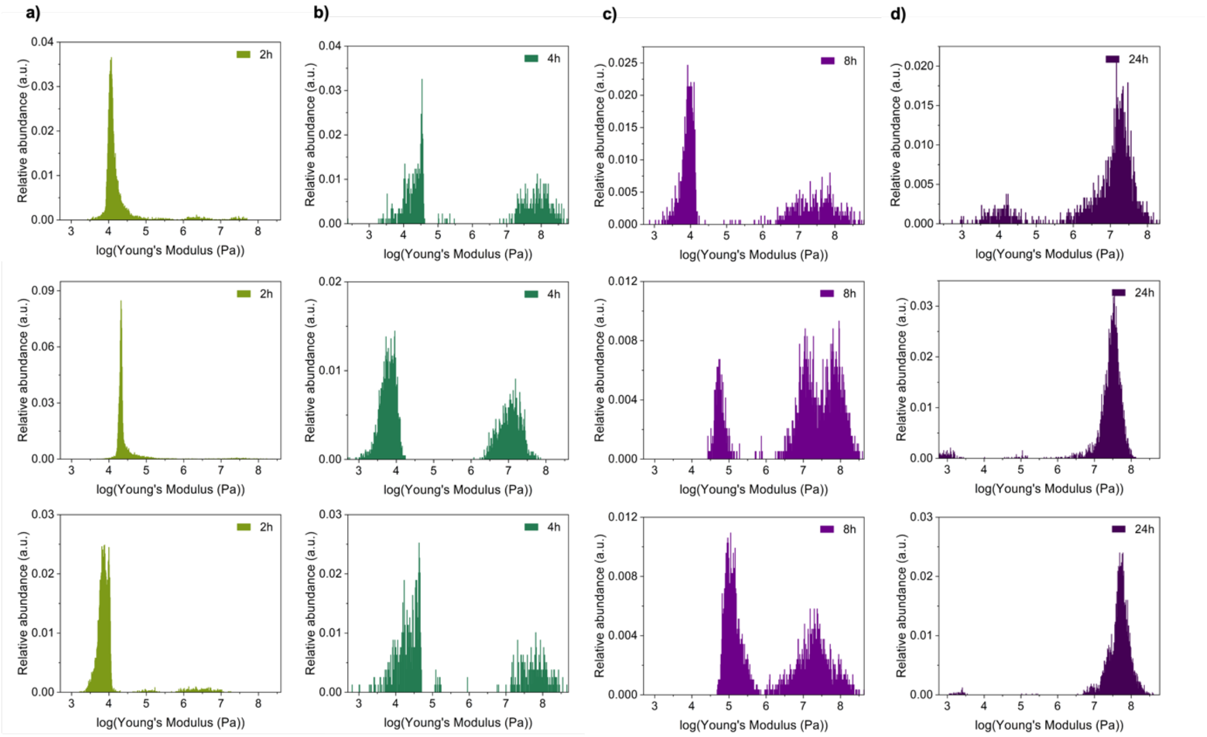

Figure S10. Phase distributions within single condensates. (a-d) Histograms of the Young's modulus distributions within single condensates are presented for t_a = 2 h (a), 4 h (b), 8 h (c) and 24 h (d). These plots reveal the presence of distinct phases: a low elastic modulus phase, an intermediate elastic modulus phase, and a high elastic modulus phase.

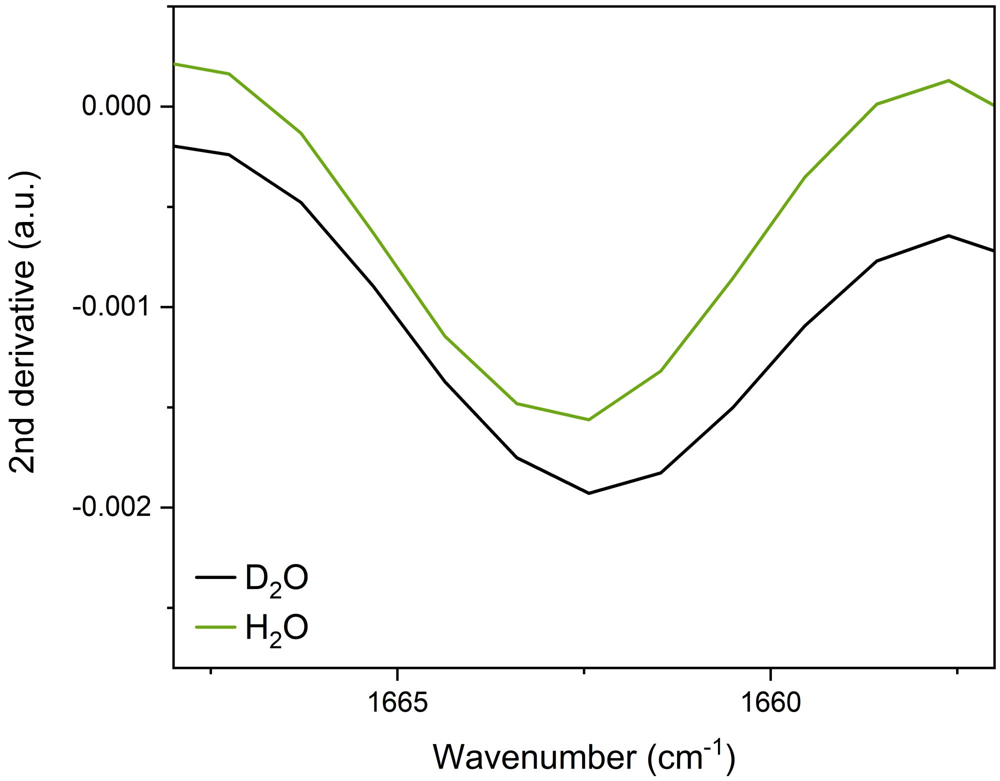

Figure S11. α-helix absorption peak for late-aged samples in H_2O and D_2O. Spectra were acquired at t_a = 8 h. The 2nd derivative is plotted in H_2O (green) and D_2O (black). The peak minimum in derivative plot in H_2O is at 1662 cm^{-1}, and we observe a downward shift of 1 cm^{-1} in D_2O. This is characteristic of α-helical secondary structure.

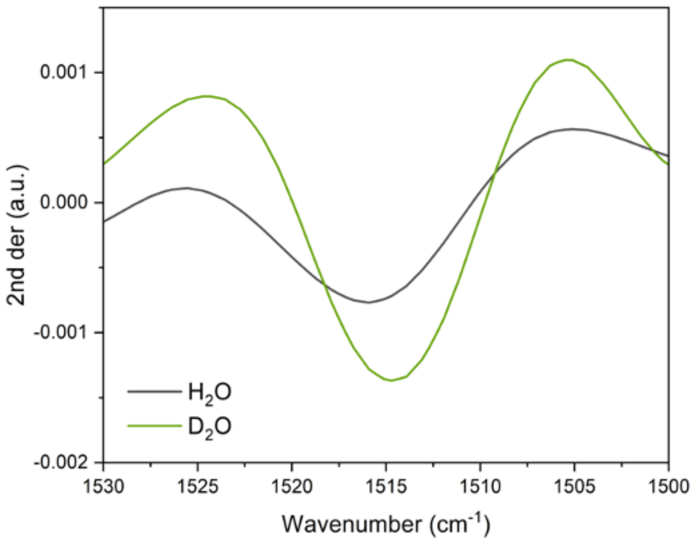

Figure S12. Tyrosine absorption peak in H_2O and D_2O. Spectra were acquired at t_a = 0 h. The 2nd derivative is plotted in H_2O (black) and D_2O (green). The peak minimum in derivative plot in H_2O is at 1517 cm^{-1}, and we observe a downward shift of 4 cm^{-1} in D_2O. This is characteristic of the tyrosine benzene ring C=C stretching vibration.

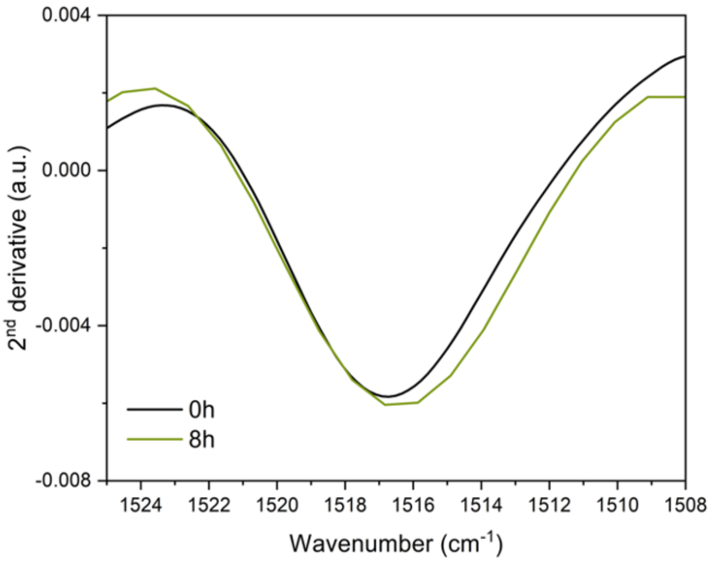

Figure S13. Tyrosine peak position at t_a = 8 h. Comparison of the tyrosine peak position for condensates at t_a = 0 and 8 h.

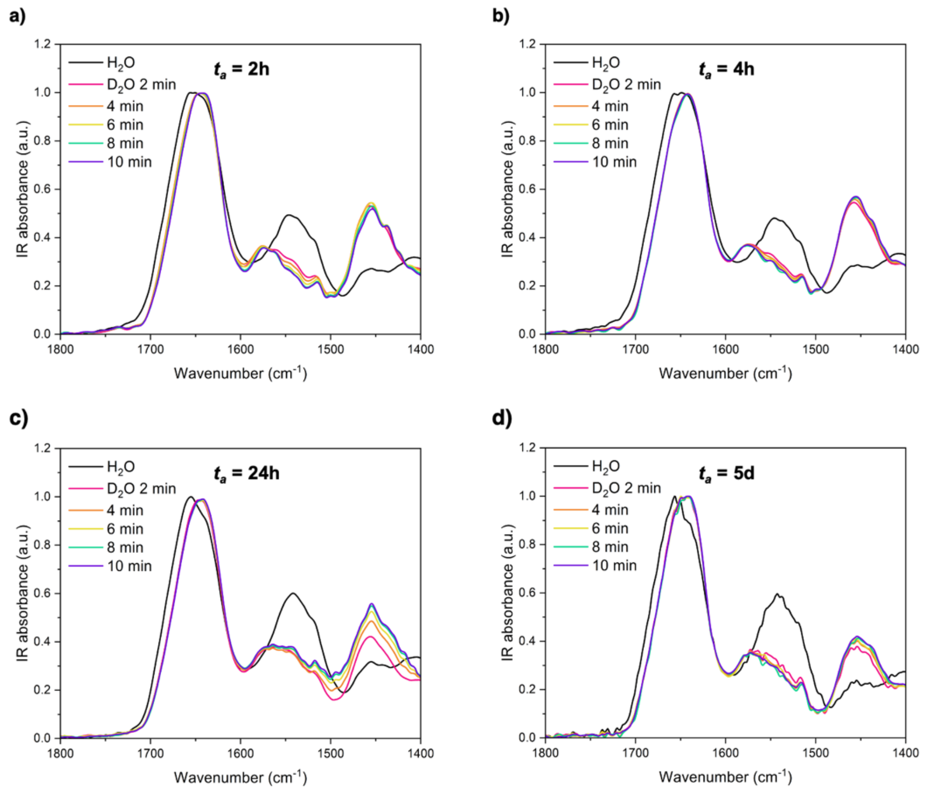

Figure S14. HDX measurements on other time points. (a-d) Spectra in H_2O (black line) and D_2O (rainbow lines) were plotted for t_a = 2 h (a), 4 h (b), 24 h (c), and 5 d (d).

Supplementary References

1. B. Derjaguin, V. Muller, Y. Toporov, Effect of contact deformation on the adhesion of elastic solids. *J. Colloid. Interface Sci*. **53**, 314–326 (1975).
2. F. S. Ruggeri, S. Vieweg, U. Cendrowska, G. Longo, A. Chiki, H. A. Lashuel, G. Dietler, Nanoscale studies link amyloid maturity with polyglutamine diseases onset. *Sci. Rep.* **6**, 1–11 (2016).
3. M. P. E. Wenger, L. Bozec, M. A. Horton, P. Mesquidaz, Mechanical properties of collagen fibrils. *Biophys. J.* **93**, 1255–1263 (2007).
4. C. A. Grant, D. J. Brockwell, S. E. Radford, N. H. Thomson, Tuning the elastic modulus of hydrated collagen fibrils. *Biophys. J.* **97**, 2985–2992 (2009).
5. N. E. Kurland, Z. Drira, V. K. Yadavalli, Measurement of nanomechanical properties of biomolecules using atomic force microscopy. *Micron*. **43**, 116–128 (2012).
6. F. W. van Tartwijk, L. C. S. Wunderlich, I. Mela, S. Makarchuk, M. A. H. Jakobs, S. Qamar, K. Franze, G. S. Kaminski Schierle, P. H. St George-Hyslop, J. Q. Lin, C. E. Holt, C. F. Kaminski, Mutation of the ALS-/FTD-Associated RNA-Binding Protein FUS Affects Axonal Development. *J. Neurosci.* **44**, 1–13 (2024).
7. S. V. Kontomaris, A. Stylianou, A. Malamou, T. Stylianopoulos, A discussion regarding the approximation of cylindrical and spherical shaped samples as half spaces in AFM nanoindentation experiments. *Mater. Res. Express*. **5** (2018), doi:10.1088/2053-1591/aad2c9.
8. B. Schuler, H. Hofmann, Single-molecule spectroscopy of protein folding dynamics-expanding scope and timescales. *Curr. Opin. Struct. Biol.* **23**, 36–47 (2013).
9. L. Jawerth, E. Fischer-Friedrich, S. Saha, J. Wang, T. Franzmann, X. Zhang, J. Sachweh, M. Ruer, M. Ijavi, S. Saha, J. Mahamid, A. A. Hyman, F. Jülicher, Protein condensates as aging Maxwell fluids. *Science (80-.).* **370**, 1317–1323 (2020).
10. M. Shamir, Y. Bar-On, R. Phillips, R. Milo, SnapShot: Timescales in Cell Biology. *Cell*. **164**, 1302-1302.e1 (2016).
11. F. S. Ruggeri, J. Charmet, T. Kartanas, Q. Peter, S. Chia, J. Habchi, C. M. Dobson, M. Vendruscolo, T. P. J. Knowles, Microfluidic deposition for resolving single-molecule protein architecture and heterogeneity. *Nat. Commun.* **9** (2018), doi:10.1038/s41467-018-06345-4.
12. T. C. T. Michaels, A. Šarić, J. Habchi, S. Chia, G. Meisl, M. Vendruscolo, C. M. Dobson, T. P. J. Knowles, Chemical Kinetics for Bridging Molecular Mechanisms and Macroscopic Measurements of Amyloid Fibril Formation. *Annu. Rev. Phys. Chem.* **69**, 273–298 (2018).
