# Supplementary material for: Nanoscale profiling of evolving intermolecular interactions in ageing FUS condensates
Source: Commun Chem. 2025 Sep 29;8:284. doi: 10.1038/s42004-025-01659-z (PMC12479745; doi:10.1038/s42004-025-01659-z)
Supplement: Supplementary file 3 — Description of Additional Supplementary Files [file 42004_2025_1659_MOESM3_ESM.pdf]

Description of Additional Supplementary Files

File name- Supplementary Data

File description - Numerical Source Data
